# Total flavonoids of *Grona styracifolia* (Osbeck) H.Ohashi and K.Ohashi capsules alleviate renal urolithiasis by modulating autophagy and endoplasmic reticulum stress

**DOI:** 10.3389/fphar.2026.1731363

**Published:** 2026-08-07

**Authors:** Zhihui Li, Ren-Ling Liu, Na Xing, Dongxiang Zheng, Xinkai Huang, Abdallah Iddy Chaurembo, Francis Chanda, Baraka Simon Mayamba, Shusheng Wang, Yingchen Zhou, Wen-Hui Deng, Han-Bin Lin, Liang Zhong

**Affiliations:** 1 The Tenth Clinical Medical College of Guangzhou University of Chinese Medicine, Zhongshan, Guangdong, China; 2 Zhongshan Hospital of Traditional Chinese Medicine, Zhongshan, China; 3 College of Chemistry and Pharmacy, Northwest A&F University, Xianyang, Shanxi, China; 4 Zhongshan Institute for Drug Discovery, Shanghai Institute of Materia Medica, Chinese Academy of Sciences, Zhongshan, Guangdong, China; 5 State Key Laboratory of Chemical Biology, Shanghai Institute of Materia Medica, Chinese Academy of Sciences, Shanghai, China; 6 University of Chinese Academy of Sciences, Beijing, China; 7 Guangdong Traditional Chinese Medicine Hospital, Guangzhou, Guangdong, China; 8 School of Pharmacy, Laboratory of Drug Discovery from Natural Resources and Industrialization, State Key Laboratory of Mechanism and Quality of Chinese Medicine, Macau University of Science and Technology, Taipa, Macau, China

**Keywords:** autophagy, calcium oxalate, endoplasmic reticulum stress, flavonoids, Grona styracifolia (Osbeck) H.Ohashi and K.Ohashi, nephrolithiasis

## Abstract

**Background:**

Calcium oxalate nephrolithiasis represents the most common form of kidney stone disease, characterized by limited therapeutic interventions and a high rate of recurrence. *Grona styracifolia* (Osbeck) H.Ohashi and K.Ohashi [syn.: *Desmodium styracifolium* (Osbeck) Merr.; Fabaceae], a traditional Chinese medicinal plant has been empirically used to treat urolithiasis; however, the mechanisms underlying its efficacy remain poorly elucidated.

**Objective:**

To investigate the anti-lithogenic mechanisms of total flavonoids of *G. styracifolia* capsules (DS) in calcium oxalate (CaOx) crystal deposition and their renal-associated injury.

**Methods:**

Sprague-Dawley rats were subjected to an ethylene glycol-induced CaOx nephrolithiasis model. The animals received DS treatment (50, 100, and 200 mg/kg) for 1 month. Thereafter, Renal histopathology was evaluated using hematoxylin and eosin staining. Furthermore, biochemical parameters such as calcium, inorganic phosphorus, creatinine, and urea nitrogen levels were assessed. Endoplasmic reticulum stress markers and autophagy-related proteins were assessed using quantitative PCR and Western blotting.

**Results:**

The DS treatment significantly reduced CaOx crystal deposition and the associated renal injury compared to untreated controls. In addition, treated animals demonstrated improved renal function, as evidenced by decreased levels of calcium, creatinine, inorganic phosphorus, and urea nitrogen. Histological examination revealed attenuated renal fibrosis and improved kidney morphology. Mechanistic investigations have indicated that the administration of DS is linked to the modulation of endoplasmic reticulum stress and autophagy, processes that are disrupted by the formation of kidney stones and urinary obstruction.

**Conclusion:**

The DS significantly mitigate CaOx nephrolithiasis by modulating endoplasmic reticulum stress and autophagy. This finding highlights its traditional therapeutic application and suggests its potential development as a phytotherapeutic agent for the prevention of kidney stones.

## 1 Introduction

Calcium oxalate (CaOx) renal stones represent the most prevalent type of urinary calculi, accounting for approximately 50% of all cases of renal urolithiasis (RU) (Tamborino et al., 2024). Renal stones are intricately associated with the supersaturation and subsequent precipitation of oxalate and calcium ions within the urinary tract. This process often leads to severe lumbar pain, hematuria, and recurrent urinary tract infections, significantly threatening human health and quality of life. Recent epidemiological studies have reported a rising global prevalence of nephrolithiasis (Wigner et al., 2021; Sorokin et al., 2017), with more cases among people residing in industrialized countries (Siener, 2021). The development of CaOx renal stones is associated with factors such as pre-existing medical conditions, familial predisposition (Howles and Thakker, 2020), and prolonged consumption of diets high in calcium and oxalate-containing foods (Ziemba and Matlaga, 2017; Alexander et al., 2022). The supersaturation of calcium and oxalate ions in urine induces a wide range of cell signaling pathways, such as cellular stress, cell death, and inflammation, which promote the pathogenesis of RU (Wu et al., 2024; Dong et al., 2025; Kumar et al., 2022).

p38 MAPK (mitogen-activated protein kinase) is a significant member of the MAPK family and plays a crucial role in fibrosis associated with chronic inflammation (Coulthard et al., 2009). Numerous studies have established a correlation between the p38 MAPK pathway and the development of CaOx renal stones (Qi et al., 2020; Guzel et al., 2021). After contact with renal tubular epithelial cells, CaOx crystals adhere to the cell surface and activate membrane receptors such as integrins (Xie et al., 2001). Furthermore, the deposition of CaOx crystals in the kidneys induces substantial generation of reactive oxygen species (ROS), subsequently leading to the phosphorylation of p38 MAPK (K and han, 2013; Xu et al., 2023; Ming et al., 2022; Yu et al., 2017). Moreover, the phosphorylation of p38 MAPK upregulates the expression of monocyte chemoattractant protein-1 (MCP-1) (Singh et al., 2021), recruiting inflammatory cells to infiltrate the tissue and exacerbate tissue damage (Wang J. et al., 2024). Damaged kidney tissue and infiltrating inflammatory cells (macrophages and lymphocytes) release large amounts of pro-inflammatory and pro-fibrotic factors, including transforming growth factor beta (TGF-β), TNF-α, and IL-1β (Mack, 2018). TGF-β1 has been widely acknowledged as a crucial mediator of kidney fibrosis, as it triggers the downstream Smad signaling pathway, which, together with the p38 MAPK signaling pathway, controls renal inflammation and fibrosis (Wu et al., 2022; Lan, 2011).

The endoplasmic reticulum (ER) is a vital organelle involved in protein biosynthesis and calcium homeostasis (Chen et al., 2023; Oakes and Papa, 2015). CaOx renal crystals disrupt ER homeostasis, leading to the accumulation of misfolded or unfolded proteins and sustained ER stress (Zeeshan et al., 2016; Han et al., 2021; Iurlaro and Munoz-Pinedo, 2016). During ER stress, biomarkers such as the chaperone protein glucose-regulated protein 78 (GRP78) and C/EBP homologous protein (CHOP) are markedly upregulated. Notably, the expression levels of these biomarkers are linked with both the duration and intensity of CaOx crystal exposure (Kaur et al., 2025; Song et al., 2020). Moreover, CaOx crystal accumulation induces endoplasmic reticulum stress (ERS), initiating crystal nucleation, aggregation, and growth during renal stone formation. Thus, regulating CaOx crystal formation and deposition in the kidneys by suppressing ER stress is a promising therapeutic strategy.

There is a complex bidirectional regulatory relationship between autophagy and renal calcium stones, which mainly affects stone formation through oxidative stress, inflammation, ER stress, and cell death pathways. Moderate autophagy alleviates inflammatory responses by clearing damaged mitochondria, lysosomes, and damage-associated molecular patterns, thereby delaying kidney stone progression (Kimura et al., 2017). High concentrations of CaOx induce excessive autophagy via the ERS-PERK pathway, leading to apoptosis, increased crystal adhesion, and kidney stone formation (Dong et al., 2022; Sun et al., 2020). Therefore, inhibiting autophagy may also have beneficial effects on renal function.

Although current therapeutic interventions, such as extracorporeal shock wave lithotripsy, can alleviate symptoms, the high recurrence rate of up to 50% within 5 years remains a significant clinical challenge (Khan et al., 2016). In light of these challenges, current research suggests that DS can significantly improve kidney function through mechanisms that remain to be fully elucidated (Zhou et al., 2018). It is, therefore, imperative to explore alternative therapeutic strategies, including DS capsules, for the treatment of urological diseases. This study employed *in vivo* rat models and molecular biology experiments to investigate the potential mechanism by which the DS inhibits CaOx crystal deposition, with a particular focus on ER stress and autophagy.

## 2 Materials and methods

### 2.1 Preparation of DS

DS Capsules (batch number: 2923002) were manufactured by Wuhan Kangle Pharmaceutical Co., Ltd., and the marketing authorization holder is Wuhan Guanggu Humanwell Bio-pharmaceutical Co., Ltd. The capsules were prepared according to the national drug registration standard (YB200402022, see Supplementary Materials, page 4). Briefly, 133 g of the total flavonoid extract from *G. styracifolia* was mixed with 37 g lactose and 30 g microcrystalline cellulose, granulated with purified water using fluid-bed granulation, dried, blended, and filled into 1000 hard gelatin capsules. Each capsule contains 133 mg of the total flavonoid extract (0.2 g per capsule). The finished product was tested in accordance with the drug registration standard and complied with all specified requirements, including dissolution, heavy metals (not more than 20 mg/kg), and microbial limits. The total flavonoid content, calculated as schaftoside, ranged from 66.5 to 87.8 mg per capsule. The contents of five major flavonoid C-glycosides (Vicenin-2, Vicenin-1, schaftoside, isovitexin, and Vicenin-3) were determined by a validated UPLC-QAMS method, with the sum ranging from 15.2 to 28.1 mg per capsule, and schaftoside alone ranging from 6.0 to 11.0 mg per capsule. The characteristic HPLC fingerprint (seven characteristic peaks) was also used for quality control (see Supplementary Materials). For administration, the capsule contents were dispersed in distilled water and diluted to the required concentration immediately before use.

### 2.2 Materials and agents

Male Sprague-Dawley rats (7–8 weeks old, body weight 180–220 g) were purchased from Zhuhai Bestest Biotechnology Co., Ltd. (Zhuhai, China). Shilitong Granules (positive control) were purchased from GUANGDONG LIFESTRONG PHARMACY CO., LTD. (National Drug Approval No. Z44023618). Ethylene glycol (EG) was procured from Shanghai Aladdin Biochemical Technology Co., Ltd, while ammonium chloride was sourced from Shanghai Macklin Biochemical Co., Ltd. All biochemical assay kits (creatinine, BUN, calcium, and phosphorus detection kits) were purchased from Shanghai Kehua Bio-Engineering Co., Ltd. Hematoxylin and eosin (H&E) staining and Masson staining were obtained from Solarbio (Beijing, China). Total RNA was extracted using an RNA extraction kit (ABclonal, Wuhan, China), while Reverse transcription and qPCR were performed using qPCR kits (ABclonal, Wuhan, China). Primers for target genes (LC3B, Beclin-1, p62, CHOP, GRP78, and β-actin) were synthesized by Sangon Biotech (Shanghai, China). Total protein was extracted using RIPA lysis buffer (Beyotime, Shanghai, China), with the protein concentration determined using a BCA kit (Beyotime). PVDF membranes (IPVH00010, Millipore, Burlington, MA, USA) were used for protein transfer. phospho-p38 (p-p38), p38, p62, TGF-β1, GRP78, LC3B, Beclin-1, MCP-1 were obtained from Affinity Biosciences (Changzhou, China). The CHOP Elisa kit was obtained from Assay Specialist Lab (Jiangsu, China).

### 2.3 DS treatment

All animals were housed under standard laboratory conditions (12 h light/dark cycle, 22 °C ± 2 °C, 50% ± 10% humidity) at the Animal Center of the Zhongshan Institute of Drug Discovery. The animal experiments in this study were approved by the Animal Ethics Committee of the Zhongshan Institute of Drug Discovery (Issue No. 2024ZSZY-LLK-272). Based on clinical observations and research background, experimental animals (7–8 weeks old, Male, N = 12) were divided into six groups. Sham group: SD rats fed with standard diet and administered normal saline (1 mL/100 g/day) via gavage; Renal Urolithiasis (RU) group: SD rats fed with standard diet and administered 1% ethylene glycol (EG) in drinking water *ad libitum* with +2% ammonium chloride solution (1 mL/100 g/day) via gavage; Positive Control (PC) group: In addition to the RU treatment, Shilitong Granules (200 mg/kg) were administered via gavage. DS+50 mg/kg group: In addition to the RU treatment, DS capsules (50 mg/kg) were administered via gavage. DS+100 mg/kg group: In addition to the RU treatment, DS total flavonoid capsules (100 mg/kg) were administered via gavage. DS+200 mg/kg group: In addition to the RU treatment, DS capsules (200 mg/kg) were administered via gavage. The doses were based on the human-equivalent dose to rats (100 mg/kg), using half as the low dose (50 mg/kg) and twice as the high dose (200 mg/kg). Following a four-week regimen of continuous treatment, urine samples were collected from the rats utilizing metabolic cages. Subsequently, blood samples were collected, and the kidneys were then harvested for further experimental analysis.

### 2.4 General biological signs

The capacity for action, behavioral status, and body weight of rats were systematically monitored. The rotarod test was employed to evaluate the behavioral status and activity levels of the rats. It measured the time it took for a rat to fall from a pole that was either rotating at varying speeds or continuously accelerating (e.g., from 4 to 40 rpm). The rotarod also measured the speed of the pole at the time of fall, and the distance traveled by the animal. The body weight of the rats was recorded weekly throughout the experiment.

### 2.5 Biochemical indicators

Following blood collection, the samples were centrifuged at 3000 rpm at 4 °C for 20 min. The resultant supernatant (serum) was then transferred to a new microcentrifuge tube and aliquoted into 100 μL portions. Urine samples were processed in a manner analogous to that of blood samples. A 100 μL volume of either serum or urine was introduced into the sample chamber of the biochemical analyzer. The predefined renal function panel, comprising serum creatinine, blood urea nitrogen (BUN), calcium, and phosphorus levels, was selected for automated analysis using the biochemical analyzer.

### 2.6 HE staining

The paraffin-embedded tissue sections were put in the oven at 60 °C for 30 min. Subsequently, paraffin was sequentially dissolved in xylene I and xylene II, each for 10 min in a fume hood. This was followed by immersion in different concentrations of alcohol, then washed accordingly. The tissue slides were stained with hematoxylin staining solution for 5 min to stain the nuclei. Subsequently, the slides were rinsed twice with distilled water, and stained with Eosin solution for 2 min and washed with distilled water for 10 s each time. Thereafter, the stained slices were dehydrated and mounted with neutral resin. The images were observed using a fluorescence microscope (BZ-X800LE). Quantification was performed using ImageJ (Version: 1.53k), and analysis was conducted at 20x magnification. Five regions were selected to measure bubble area, and the ratio of the bubble area to the total area was calculated.

### 2.7 Masson staining

The paraffin-embedded tissues were subjected to a similar dewaxing procedure as the HE staining. Thereafter, the tissue slides were stained with Weigert’s Iron Hematoxylin solution for 5 min. Subsequently, the slides were rinsed thoroughly and stained with Masson composite staining solution for 5 min. The slides were then dipped in Phosphomolybdic Acid solution and later Aniline Blue solution. Thereafter, they were washed briefly in distilled water and quenched with a weak Acetic Acid solution (e.g., 1%). The stained slices were dehydrated and mounted with neutral resin. The images were observed under a fluorescence microscope (BZ-X800LE), while quantification was performed using ImageJ (Version: 1.53k), and analysis was conducted at 20x magnification. Five regions were selected to measure the fibrosis area, and the ratio of the fibrosis area to the total area was calculated.

### 2.8 Real-time quantitative PCR (RT-qPCR)

The expression levels of mRNA for autophagy-related factors (LC3B, Beclin-1, and p62) and ERS-related factors (CHOP and GRP78) in rat kidney tissues were quantified using RT-qPCR. Total RNA was extracted using TRIzol reagent according to the manufacturer’s protocol, and cDNA was synthesized with a reverse transcription synthesis kit. qRT-PCR was then performed using SYBR Green Fast qPCR Mix. The primer sequences are provided in Supplementary Table S1.

### 2.9 Western blot

The total proteins of the kidney samples were extracted using the radioimmunoprecipitation assay RIPA and quantified using BCA. For western assay, the total proteins were separated by polyacrylamide gel electrophoresis, and transferred to PVDF membrane, which were blocked with 5% skim milk, washed, and incubated with primary antibodies (phospho-p38 (p-p38), p38, p62, TGF-β1, GRP78, LC3B, Beclin-1, MCP-1) for 12 h. Subsequently, membranes were washed and incubated with secondary antibodies for 2 h, followed by chemiluminescence using Amersham Image Quant™ 800 (Danaher Corporation, United States). The ImageJ software was used to calculate the grayscale values of the proteins, and GAPDH was used as a reference for normalization.

### 2.10 Statistical analysis

All experimental data were statistically analyzed using the GraphPad Prism software (version 9.0). The results are expressed as the mean ± standard deviation (mean ± SD). Comparisons among multiple groups were performed using one-way analysis of variance (One-Way ANOVA), with *P* < 0.05 considered statistically significant.

## 3 Results

### 3.1 DS improved rat general biological activities

**FIGURE 1:**
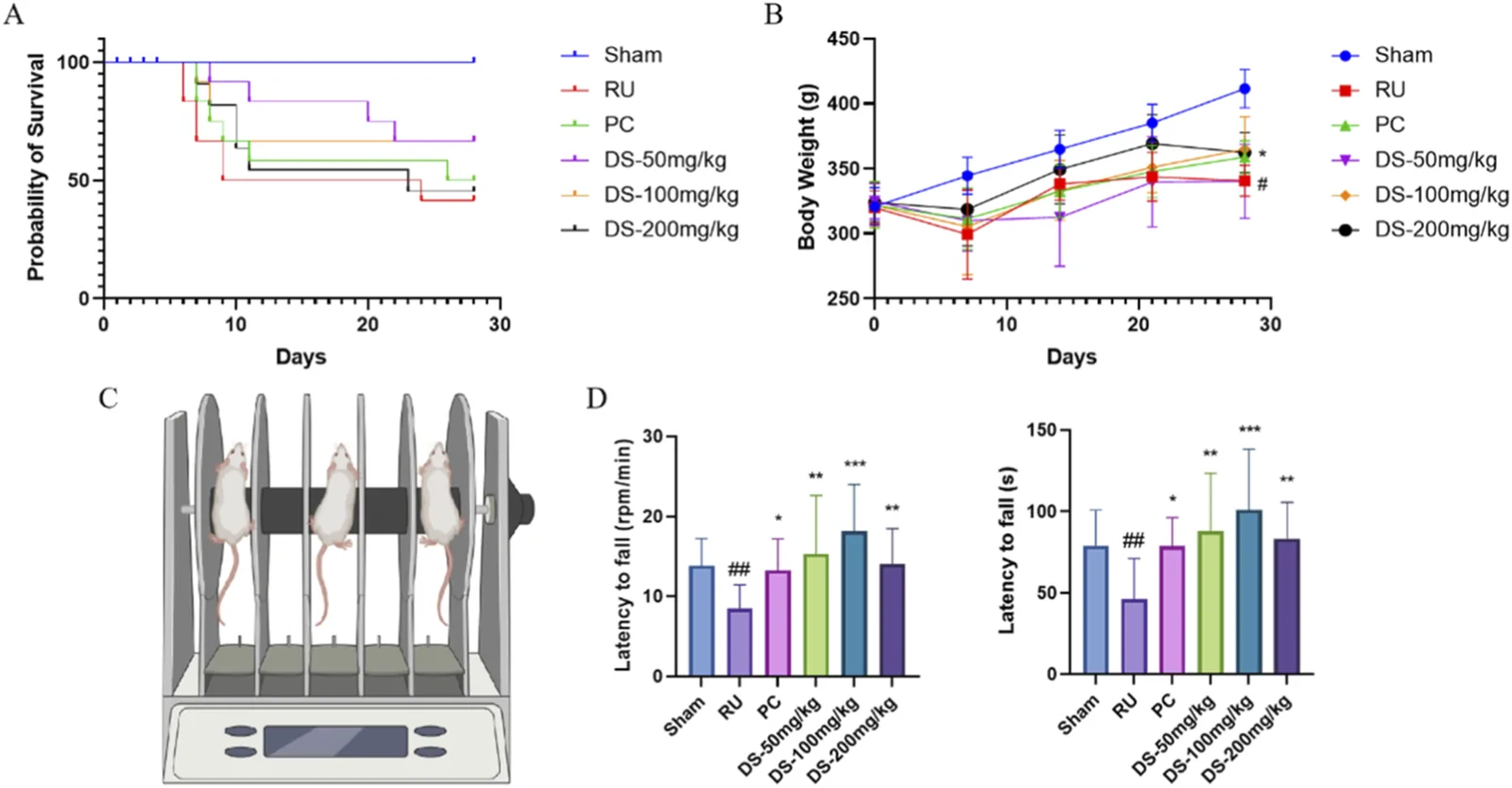
General biological signs **(A)** Rat survival curve. **(B)** Body weight changes in the rats. **(C)** Schematic representation of the rotarod test. **(D)** Speed of the rat when falling and time to fall. The results are expressed as mean ± SD, n ≥ 6; ^#^, compared with the sham group, P < 0.05; ^##^, compared with the sham group, *P* < 0.01; *, Compared with the RU group, *P* < 0.05; **, Compared with the RU group, *P* < 0.01; ***, Compared with the RU group, *P* < 0.001.

In this study, we employed 1% EG and 2% ammonium chloride solutions to induce CaOx nephrolithiasis and assessed the specific role of DS. Following oral gavage administration, body weight and mortality were systematically recorded. Notably, sham rats in the RU group exhibited significant mortality, while rats treated with DS exhibited enhanced survival rates (Figure 1A). Furthermore, less body weight was recorded in rats treated with 200 mg/kg DS (Figure 1B). Interestingly, rats in the RU group exhibited a significant decline in motor function, whereas those treated with DS showed improved motor function, as evidenced by the prolonged duration on the rotarod and enhanced adaptation to its speed (Figures 1C,D). These findings indicate that DS treatment protects against CaOx nephrolithiasis and enhances motor function in rats.

### 3.2 DS reduces renal CaOx stone formation

**FIGURE 2:**
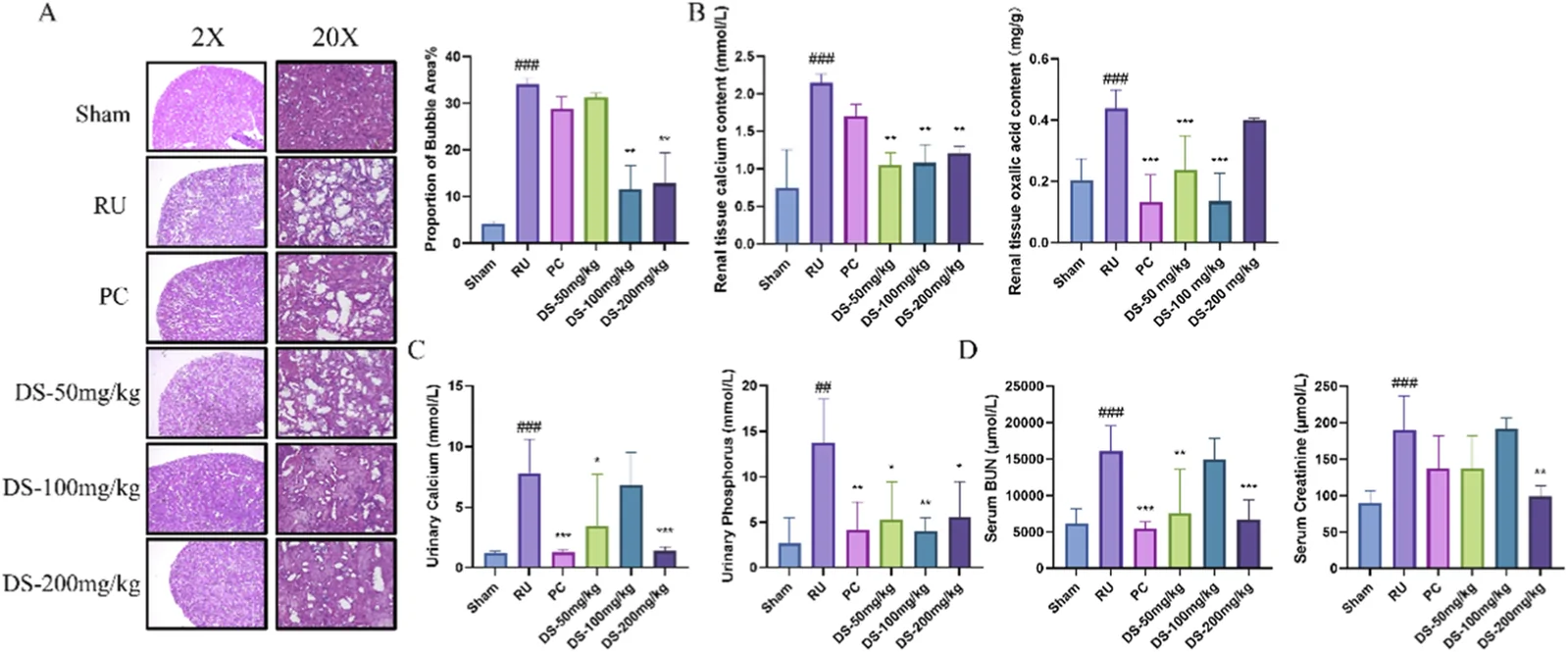
Reduction of urinary stone deposition **(A)** Hematoxylin and eosin (HE) staining; **(B)** Renal tissue calcium and oxalic acid content; **(C)** Urinary biomarkers; **(D)** Serum biomarkers. The results are expressed as mean ± SD, n ≥ 6; ^##^, compared with the sham group, *P* < 0.01; ^###^, compared with the sham group, *P* < 0.001; *, Compared with the RU group, *P* < 0.05; **, Compared with the RU group, *P* < 0.01; ***, Compared with the RU group, *P* < 0.001.

To validate whether the observed improvement in rat biological activities was linked to a reduction in CaOx renal crystals deposition, we performed HE staining of kidney sections for histological observation and quantitative statistical analysis. The results demonstrated extensive crystal accumulation in the, EG induced model group. Notably, DS treatment significantly alleviated crystal formation and deposition in rat renal tissues (Figure 2A). Furthermore, we measured oxalate and calcium levels in renal tissue. Under, EG induction, both were significantly elevated, indicating that the renal crystals were characterized by CaOx. Notably, DS treatment markedly decreased both oxalate and calcium levels (Figure 2B). Similarly, the renal tissue calcium content was significantly decreased in the DS-treated group (Figure 2C). In addition, urinary biomarkers (calcium, phosphorus) and kidney function biomarkers (BUN, and creatinine), were markedly reduced after oral DS administration compared to the RU group (Figures 2B,D). The decrease in these biochemical markers contributes to the reduced formation of CaOx crystals.

### 3.3 DS mitigates CaOx-induced kidney injury

**FIGURE 3:**
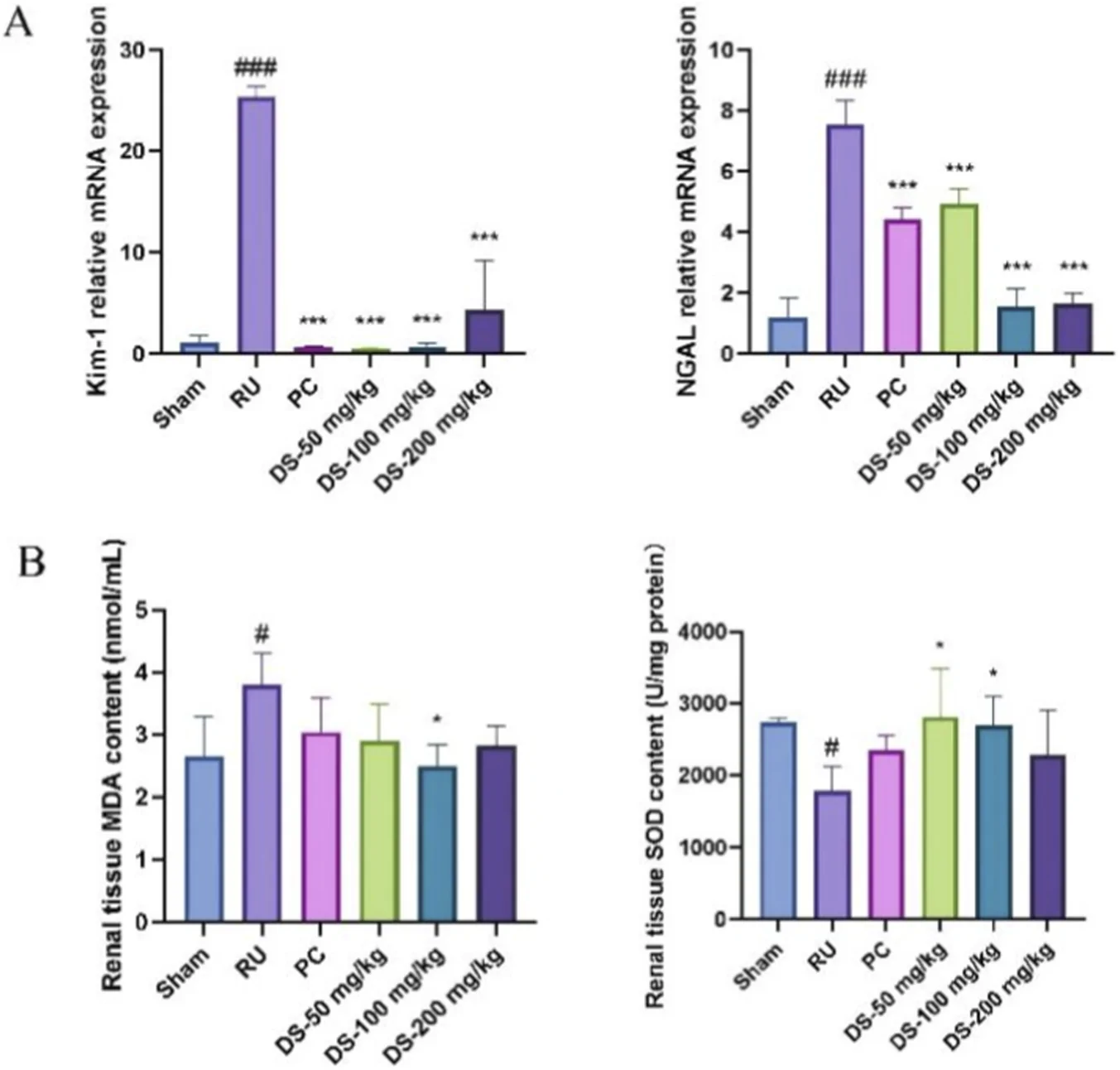
DS mitigates CaOx-induced kidney injury **(A)** Kidney injury markers; **(B)** Oxidative stress markers. The results are expressed as mean ± SD, n ≥ 6; ^#^, compared with the sham group, *P* < 0.05; ^###^, compared with the sham group, *P* < 0.001; *, Compared with the RU group, *P* < 0.05; ***, Compared with the RU group, *P* < 0.001.

To further evaluate the protective effects of DS against CaOx crystal-induced renal tubular injury, kidney injury markers and oxidative stress indicators were examined. As shown in Figure 3A, the RU group demonstrated elevated kidney injury markers (such as Kim-1 and NGAL) compared with the sham group, indicating severe renal tubular damage and impaired renal function. Treatment with DS at different doses effectively reduced these kidney injury biomarkers, indicating a protective effect on renal tissue. Meanwhile, oxidative stress was markedly aggravated in the RU model group, as evidenced by increased MDA levels and decreased SOD activity (Figure 3B). These changes suggest enhanced lipid peroxidation and weakened antioxidant capacity. In contrast, DS treatment decreased MDA levels and increased SOD activity. This suggests that DS enhances antioxidant capacity and alleviates oxidative stress in rats with CaOx-induced nephrolithiasis.

### 3.4 DS administration inhibits inflammation and fibrosis in the CaOx crystal deposition model

**FIGURE 4:**
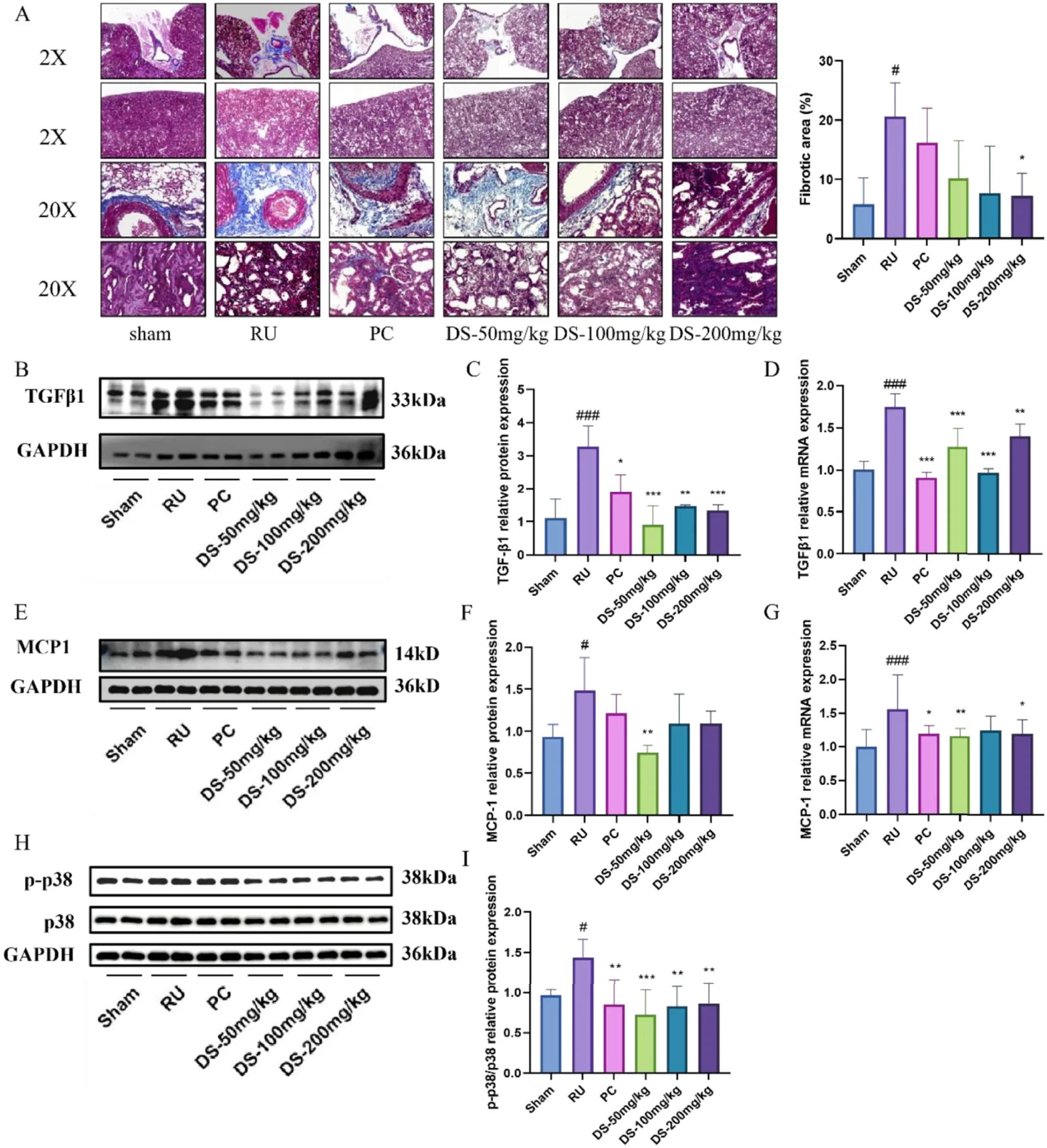
The effect of DS on inflammation and fibrosis in the calcium oxalate crystal deposition model. **(A)** Masson staining; **(B)** Representative immunoblotting and graphic presentation of TGF-β1; **(C)** Relative protein level of TGF-β1; **(D)** mRNA level of TGF-β1; **(E)** Representative immunoblotting and graphic presentation of MCP-1; **(F)** Relative protein level of MCP-1; **(G)** mRNA level of MCP-1; **(H)** Representative immunoblotting and graphic presentation of p-p38/p38; **(I)** Relative protein level of p-p38/p38. The results are expressed as mean ± SD, n ≥ 6; ^#^, compared with the sham group, *P* < 0.05; ^###^, compared with the sham group, *P* < 0.001; *, Compared with the RU group, *P* < 0.05; **, Compared with the RU group, *P* < 0.01; ***, Compared with the RU group, *P* < 0.001.

During kidney stone formation, CaOx crystals damage renal tubular epithelial cells, thereby initiating an innate immune response that leads to inflammation, marked by fibrosis. Compared with the sham group, renal fibrosis was aggravated in the RU, which was effectively reduced by DS treatment (Figure 4A). Considering that inflammation and TGF-β activation play pivotal roles in kidney stone pathogenesis, we evaluated the effects of DS on inflammation and fibrosis. The results showed elevated protein levels of p-38/p38, MCP-1, and TGF-β in the RU group, which were suppressed by DS treatment (Figures 4B,C,E,F,H,I), with the mRNA levels of MCP-1 and TGF-β following the same trend (Figures 4D,G). These results indicate that DS treatment alleviated kidney fibrosis and inflammation in rats with CaOx renal stones.

### 3.5 DS alleviates kidney injury through inhibition of autophagy

**FIGURE 5:**
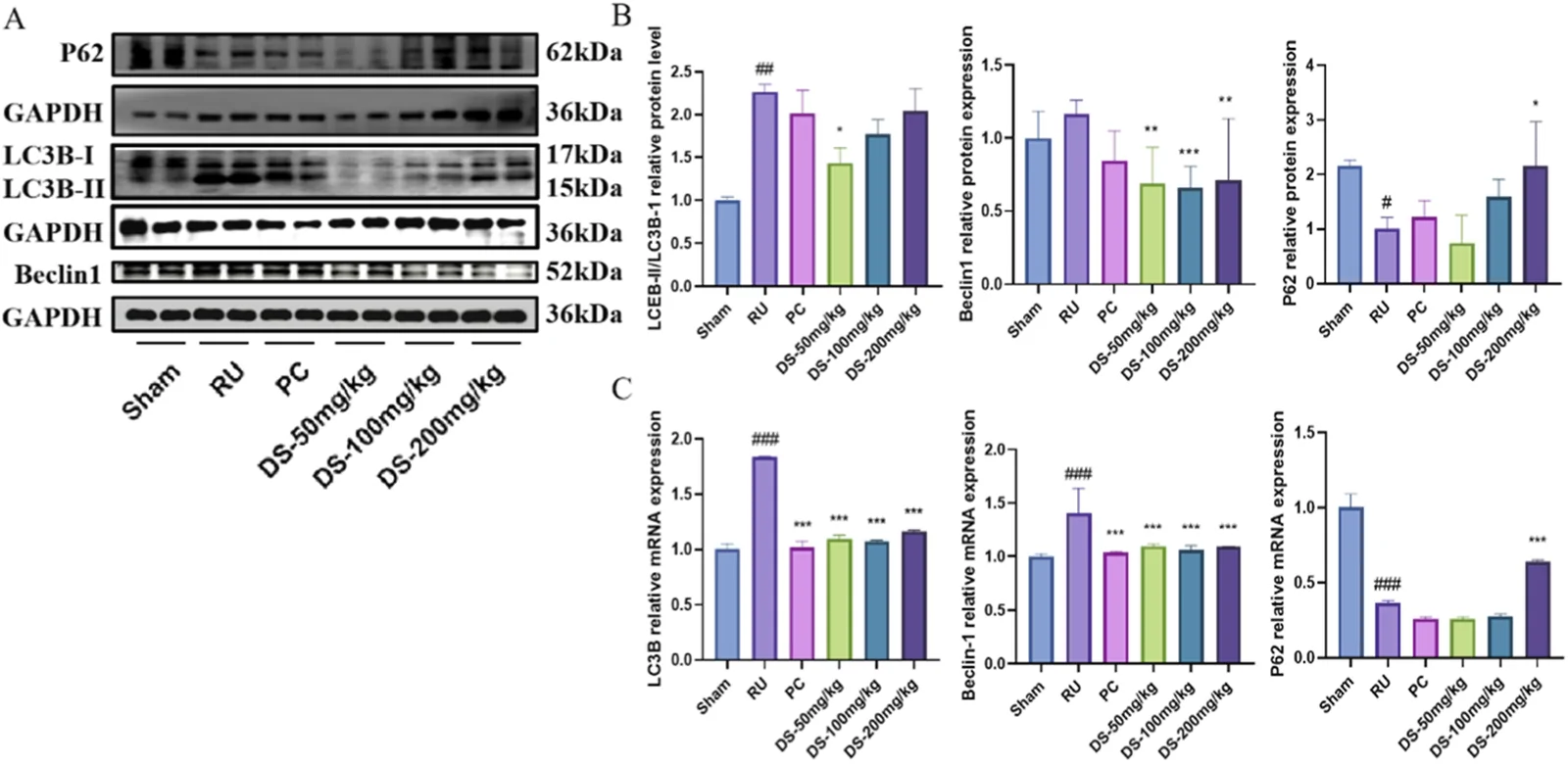
DS alleviates kidney injury through inhibition of autophagy **(A)** Representative immunoblotting and graphic presentation of LC3B, Beclin-1 and P62; **(B)** The relative protein level of LC3B, Beclin-1 and P62; **(C)** The mRNA level of LC3B, Beclin-1 and P62. The results are expressed as mean ± SD, n ≥ 6; ^#^, Compared with the sham group, *P* < 0.05; ^###^, Compared with the sham group, *P* < 0.001; *, Compared with the RU group, *P* < 0.05; **, Compared with the RU group, *P* < 0.01; ***, Compared with the RU group, *P* < 0.001.

Severe CaOx crystal exposure leads to excessive activation of autophagy, triggering lysosomal leakage and mitochondrial dysfunction. This exacerbates cell death and further promotes CaOx crystal nucleation and deposition (Li et al., 2022). To assess whether DS treatment attenuates pathological autophagy in CaOx renal stone tissues, the expression of the critical autophagic biomarkers such as LC3B, Beclin-1, and P62 were evaluated using Western blotting Figure 5A. In the RU group, autophagy-related proteins were markedly increased, whereas DS treatment significantly reversed this expression (Figures 5B,C). These results indicate that DS treatment inhibits CaOx-induced pathological autophagy.

### 3.6 DS treatment alleviates ERS in the kidney of CaOx crystal deposition

**FIGURE 6:**
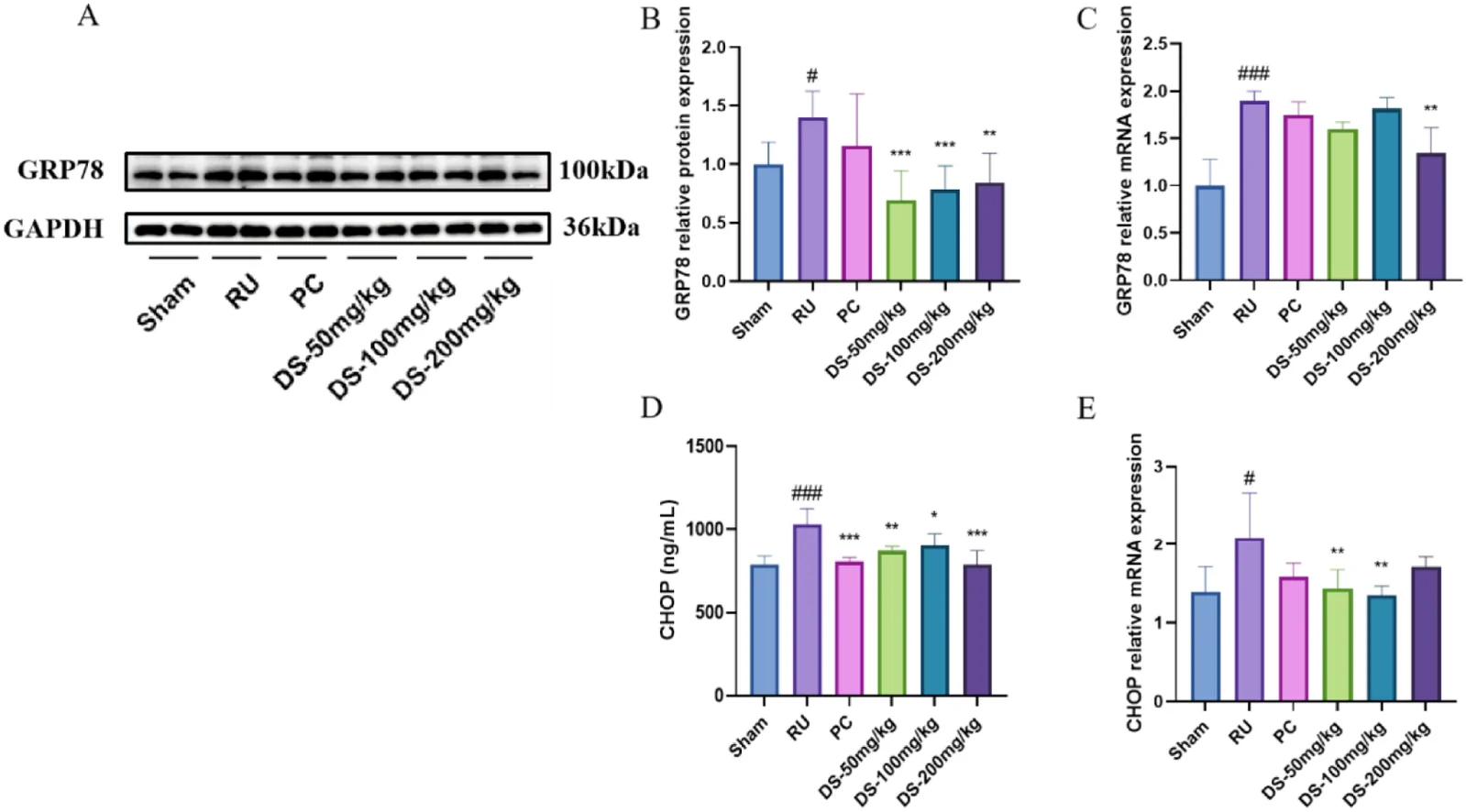
DS treatment alleviates ERS in the kidney of calcium oxalate crystal deposition **(A)** Representative immunoblotting and graphic presentation of GRP78; **(B)** The relative protein level of GRP78; **(C)** The mRNA level of GRP78; **(D)** The concentration of serum CHOP; **(E)** The mRNA level of CHOP. The results are expressed as mean ± SD, n ≥ 6; ^#^, Compared with the sham group, *P* < 0.05; ^##^, Compared with the sham group, *P* < 0.01; ^###^, Compared with the sham group, *P* < 0.001; *, Compared with the RU group, *P* < 0.05; **, Compared with the RU group, *P* < 0.01; ***, Compared with the RU group, *P* < 0.001.

To evaluate the therapeutic effect of DS treatment on ER stress in the kidney stone model, we examined the relevant markers accordingly (Figures 6A,B,D). The results showed that when CaOx kidney stones were formed, the mRNA expression levels of CHOP and GRP78 were markedly elevated in the kidneys forming CaOx stones, and these expression levels were reduced after DS treatment (Figures 6C,E). Moreover, DS treatment downregulated GRP78 protein and serum CHOP levels. Thus, DS treatment alleviated ER stress in the kidneys of mice with CaOx crystal deposition.

## 4 Discussion

The rising global incidence of renal stones underscores the need to explore innovative therapeutic approaches that target the pathogenesis rather than merely managing symptoms. In this study, we suggested that the total flavonoid content of DS capsules significantly reduced CaOx crystal deposition in rats by modulating autophagy and suppressing ER stress.

The formation of CaOx crystals into stones can irritate the renal system and induce renal colic, thereby decreasing the capacity for physical activity. As such, the rotarod test was used to assess the behavioral status and activity levels of the rats (Miranda et al., 2015; Xiao et al., 2024). Our findings indicate that DS treatment significantly enhanced motor function in rats with CaOx renal stones, mitigated pain-induced appetite loss, and decreased body weight loss, suggesting systemic benefits that extend beyond the renal protection.

Abnormalities in ion concentrations play an important role in kidney stone development (Dong et al., 2024). Calcium and inorganic phosphate account for the most common type of calculi (Sakhaee et al., 2012). DS effectively reduced renal CaOx crystal deposition and decreased the calcium content in the kidney tissue. In the present study, this was substantiated by the observed reduction in the urinary levels of calcium, phosphorus, urea nitrogen, and creatinine. These findings suggest that DS restores urinary biochemical homeostasis, which is essential for preventing crystal nucleation driven by urine supersaturation. Moreover, these findings not only align with but also expand upon previous research on DS extracts. For instance, Zhou et al. (2018) reported that DS mitigate hydroxyl-proline-induced CaOx urolithiasis primarily through their antioxidant properties and urine alkalinizing effects, which help reduce crystal deposition and the expression of MCP-1/TGF-β. Our study supports these anti-stone and anti-inflammatory effects while offering mechanistic insight.

Kidney stones induce ER stress in renal cells, as shown by the upregulation of CHOP and GRP78 mRNA levels (Ke et al., 2024). DS treatment effectively downregulated these biomarkers and mitigated associated cellular damage. Concurrently, DS reduced the expression of autophagy-related genes and proteins (LC3B and Beclin-1), suggesting the modulation of stress-induced autophagic flux (Liu et al., 2018). Autophagy plays a dual role in nephrolithiasis. On one hand, moderate autophagy helps maintain cellular homeostasis by clearing away damaged organelles, misfolded proteins, and impaired mitochondria. On the other hand, excessive autophagy, triggered by CaOx crystals through ROS/ER stress, leads to lysosomal leakage, mitochondrial dysfunction, renal tubular epithelial cell death, and increased crystal adhesion and deposition, ultimately promoting stone formation and disease progression (Sun et al., 2020; Kang et al., 2020). Other studies have revealed elevated autophagy markers, such as LC3B and Beclin-1, in the EG-induced nephrolithiasis model. Notably, DS treatment suppresses these key autophagy markers, which is accompanied by reduced crystal deposition and improved renal histology. This suggests that inhibiting maladaptive autophagy serves as an important protective mechanism, consistent with previous findings (Liu et al., 2025; Xu et al., 2025; Chen et al., 2025).

DS suppressed the expression levels of MCP-1 and TGF-β, which align with the role of P38 MAPK in inflammation: CaOx crystals activate P38 via ROS signaling, elevating MCP-1 levels and promoting leukocyte infiltration (Huang et al., 2014; Kobayashi et al., 2017). DS disrupts this inflammatory cascade and mitigates crystal-induced tubular damage and fibrosis by inhibiting P38 phosphorylation. Similarly, it was demonstrated in COM-treated HK-2 cells that DS flavones inhibit p38/MAPK-mediated KIM-1 upregulation, thereby reducing apoptosis and excessive autophagy (Xie et al., 2018). This is consistent with other studies, which revealed that DS and its main flavones (luteolin, apigenin, and genistein) exert protective effects against oxalate-induced kidney injury primarily through inhibition of p38 MAPK phosphorylation and CTSD expression (Hou et al., 2018). Our animal data support this pathway; however, we used DS instead of the whole extract, thereby excluding the effects of other active components. This further confirms that are the primary bioactive substances mediating the therapeutic effects of DS. Additionally, we associated p38 inhibition with the attenuation of ER stress and the mitigation of fibrosis, offering new insights into the multitarget mechanisms of DS total flavones against crystal-induced kidney injury.

p38 MAPK plays a crucial role in the cellular stress response, linking ER stress and autophagy. Phosphorylated and activated by ER stress via enhancement of CHOP and GRP78 expression, p38 MAPK induces excessive autophagy through modulation of Beclin-1 and LC3B (Li et al., 2017; Shimada et al., 2011). Our results indicated that DS significantly reduce p38 phosphorylation, accompanied by concurrent downregulation of ER stress markers (GRP78, CHOP) and autophagy markers (LC3B, Beclin-1), as well as reduced crystal deposition and renal injury. Consistent with previous studies, it is demonstrated that nano-COM crystals induce ER stress in HK-2 cells, characterized by elevated ER Ca^2+^ levels, increased CHOP and Caspase-12 expression, and concurrent activation of p38 MAPK; inhibition of this pathway significantly attenuated crystal-induced apoptosis and adhesion (He et al., 2024). Similarly, CaOx crystal-induced autophagy in renal tubular cells is regulated via the SIRT1/AKT/p38 signaling axis, where modulation of p38 contributes to autophagic flux control (Jing et al., 2022). Furthermore, autophagy activation exacerbates oxalate-induced oxidative injury and directly promotes p38 phosphorylation, while chloroquine (an autophagy antagonist) or Beclin-1 knockdown markedly suppresses p-p38 levels, mitochondrial damage, and CaOx crystal deposition *in vitro* and in EG-induced rat models (Duan et al., 2018). This multitarget efficacy of DS is likely attributable to its ability to inhibit p38 phosphorylation, thereby disrupting these interconnected pathways.

Additionally, studies on DS polysaccharides highlight complementary mechanisms, such as reduced crystal adhesion/endocytosis and inhibition of the TGF-β/Smad pathway (Wang Z. et al., 2024; Yu et al., 2025). These findings suggest that DS provides protective effects through a multi-pathway approach, highlighting an advantage over single-target drugs.

## 5 Limitations

While the current study demonstrates that DS offer protective effects against EG-induced CaOx nephrolithiasis primarily by inhibiting ER stress and autophagy, it is important to acknowledge limitations. Initially, the intervention utilized a total flavonoid fraction instead of a specific active component. Consequently, it remains uncertain which flavonoid constituent plays the primary role in the therapeutic effects. As such, future research involving isolated single compounds is essential to identify the main active ingredients and potential synergistic interactions. Furthermore, we cannot rule out the possibility that total flavonoids may offer superior therapeutic efficacy compared to any single compound, owing to potential synergistic interactions. Additionally, while this study concentrated on CaOx nephrolithiasis, the identification of renal stone composition was somewhat preliminary. We mainly relied on HE staining, renal tissue calcium and oxalate content, and biochemical indicators. Thus, the study’s conclusions are primarily applicable to CaOx stones and may not be generalizable to other stone compositions. Furthermore, this study was conducted in male SD rats using a single, EG and ammonium chloride induction model over 4-week. The direct molecular target of DS remains unidentified, as the present study only investigated the regulatory effects on downstream signaling pathways (p38 MAPK, ER stress, and autophagy). Our study suggests that p38 MAPK may serve as a key upstream signaling molecule through which DS regulate ER stress and autophagy. However, the current evidence is primarily based on correlative analyses and pathway inhibition observations, lacking direct causal validation. Future studies should employ specific p38 inhibitors (SB203580) or p38 overexpression (using adenoviral or lentiviral vectors) to intervene and observe their effects on EG-induced ER stress, autophagic flux, and nephrolithiasis formation. This will help further clarify the central role of p38 MAPK in the protective effects of DS and elucidate the upstream and downstream causal relationships. Finally, these findings require further validation in additional animal models (including female animals), and other stone-induction methods. Moreover, DS, as a Chinese patent drug, collecting clinical data from patients using the drug in the future will greatly facilitate the identification of its direct molecular targets.

## 6 Conclusion

DS treatment effectively inhibited renal calculi formation and consequent renal damage. This therapeutic effect is likely attributable to the mitigation of ER stress and autophagy, potentially mediated through p38 MAPK regulation. Thus, DS treatment may be a viable therapeutic strategy for the treatment of nephrolithiasis.

## Data Availability

The original contributions presented in the study are included in the article/supplementary material, further inquiries can be directed to the corresponding authors.
